# Influence of Age, Gender and Education Level on Executive Functions and Functioning in People with Stroke

**DOI:** 10.3390/biomedicines11061603

**Published:** 2023-06-01

**Authors:** Patricia Sánchez-Herrera-Baeza, Roberto Cano-de-la-Cuerda, Sergio Serrada-Tejeda, Diego Fernández-Vázquez, Víctor Navarro-López, Carlos González-Alted, Juan Carlos Miangolarra-Page

**Affiliations:** 1 Department of Physical Therapy, Occupational Therapy, Rehabilitation, and Physical Medicine, Faculty of Health Sciences, Rey Juan Carlos University, 28922 Madrid, Spain; patricia.sanchezherrera@urjc.es (P.S.-H.-B.); sergio.tejeda@urjc.es (S.S.-T.); diego.fernandez@urjc.es (D.F.-V.); victor.navarro@urjc.es (V.N.-L.); juan.miangolarra@urjc.es (J.C.M.-P.); 2 Centro de Referencia Estatal a la Atención del Daño Cerebral (CEADAC), C/del Río Bullaque, 1, 28034 Madrid, Spain

**Keywords:** activities of daily living, age, education level, executive functions, gender, stroke

## Abstract

Background: Alterations in mental functions are among the most frequent manifestations of stroke that have a direct impact on the patient’s functionality. The objective of this study was to analyze the relationship of sociodemographic variables with the executive functions (EFs) of participants with right middle cerebral artery (MCA) stroke. Methods: A cross-sectional observational case-control study was conducted at the State Center for Brain Damage in Madrid, Spain. Fifty-eight subjects were recruited and divided into two groups. Each participant was administered the following: the FIM+FAM Functional Assessment Measure, the Lawton and Brody scale, The Trail-Making Test, the Zoo Map Test and the Hanoi Tower. Results: Statistically significant differences (*p* < 0.05) were identified between participants with ischemic stroke and control in functional and EF functions, as well as between participants with hemorrhagic stroke and control. No statistically significant differences were found in the experimental group between subjects who had sustained ischemic and hemorrhagic stroke. No significant associations were identified between the variables age, gender and education level in relation to functionality and executive functions (*p* > 0.05) in people with stroke. Conclusion: People who have suffered a right cerebral artery stroke have deficiencies in the EFS, resulting in poorer performance of the activity of daily living, compared to healthy subjects of the same age, gender and education level. In the correlational analysis of the stroke participants, no significant associations were identified between the variables gender, age and education level in relation to functionality and EF.

## 1. Introduction

Stroke is one of the leading causes of morbidity and mortality in developed countries and, according to data from the World Health Organization, is the third leading cause of death in the Western world, the leading cause of physical disability in adults and the second leading cause of dementia [1]. The incidence of stroke varies between countries and settings, and increases with age due to changes in the cerebral vasculature and the high presence of multimorbidity in older people [2].

The incidence rate of first stroke in adults aged 45 to 84 years is between 100 and 600 cases per 100,000 inhabitants/year [3]. In industrialized countries, it is responsible for more than 10% of all deaths and 88% of all deaths occurring in people over 65 years of age, with an average age of around 75 years [4]. In Spain, it is estimated that the prevalence of stroke is 1.7%, being the second cause of global death, the third in men and the first in women [5]. 

Unlike other vascular diseases, stroke is a heterogeneous entity, caused by various pathophysiological mechanisms. Approximately 85% of strokes are ischemic and are caused by small vessel brain disease, cardioembolism and thromboembolism related to atherosclerosis of large arteries. About 15% of strokes are caused by intracerebral hemorrhage, which can be deep or lobar; 80% of these are the result of small vessel brain diseases (deep perforating artery disease, cerebral amyloid angiopathy) [6]. The factors that determine the severity of a stroke, as well as its sequelae, are the area and extent of the brain affected, the time it takes to restore blood supply to the injured areas and the capacity of the intact areas of the brain to replace or compensate for the injured functions [7].

Alterations in mental functions, both cognitive and affective, are some of the most common and, at the same time, least recognized manifestations of stroke. Recent research indicates a prevalence of dementia of up to 20% and mild cognitive impairment (MCI) of 38%, and a prevalence of depression and anxiety reaching 30% and 20–25%, respectively. These, in turn, have a direct impact on the patient’s functionality [8,9].

All the activities we perform in our daily life require the use of executive functions (EFs), the mental processes that allow us to focus attention on specific tasks, participate in successful problem solving and plan for the future [10]. EFs are best described as a complex set of cognitive skills that include working memory, inhibitory control, planning, reasoning, problem solving and cognitive flexibility. For instance, cognitive flexibility plays an important role in our everyday life, since it allows an individual to work efficiently to disconnect from a previous task, configure a new set of responses and implement this new set of responses in another task, in such a way that allows one to adapt to new, changing or unplanned events [11,12].

The development of EF is linked to that of the prefrontal cortex and depends on the maturity and integrity of other regions, both cortical and subcortical [13]. Scientific literature has shown that stroke is associated with impaired executive functions and some authors have reported a significant short-term decline in cognitive function followed by an accelerated rate of post-stroke cognitive decline, and other studies report only a short-term decline in cognitive function without long-term acceleration of cognitive decline [14]. This impairment in EFs may affect the functionality of the individual, reducing his or her level of independence, both at the level of basic activities of daily living (ADLs) and instrumental activities of daily living [15]. In line with these studies, different investigations have tried to identify factors related to the functional recovery of the stroke patient, such as age or sex. In this regard, in comparison to male patients [16,17], recent studies [14] have identified that in women, stroke is associated with a short-term acceleration of cognitive impairment and the presence of greater physical limitations in the performance of activities of daily living, due to their life expectancy and age at the time of stroke [18]. These findings are in line with [14], in which in a nationally representative cohort of community-dwelling adults in the US, they found, after controlling for pre-stroke cognitive measures, that females, in contrast to males, experience post-stroke cognitive deficits, particularly during the early post-stroke period. In addition, recent studies in patients with ischemic stroke have identified that cognitive status and executive functions showed lower levels than would be expected for their age and education level [19,20].

Considering the scarce number of studies, improving the understanding and comprehension of the relationship of sociodemographic factors such as age, gender and education level on executive functions and ADL independence is necessary. For this purpose, the aim of this study was to analyze the relationship of sociodemographic variables with EF and the levels of functionality and independence of persons who have suffered a right cerebral artery stroke in comparison with a healthy population. In addition, we analyzed the data according to the type of stroke: ischemic or hemorrhagic. Our initial hypothesis was that healthy subjects presented better scores in EF compared to stroke participants. Further, we considered that EF did not present differences between ischemic and hemorrhagic participants. Finally, we hypothesized that age, gender and education level present a relationship with EF in people with stroke.

## 2. Materials and Methods

An observational cross-sectional case control study was conducted at the State Center for Brain Injury in Madrid, Spain. We followed the Strengthening the Reporting of Observational Studies in Epidemiology guidelines [21] to standardize the reporting of this research. 

### 2.1. Participants

Sixty subjects were finally recruited and divided into two groups. The experimental group consisted of 30 participants with right middle cerebral artery (MCA) stroke and with EF impairment who were undergoing rehabilitation. The control group consisted of 30 healthy subjects matched for age, gender and education level.

The inclusion criteria for the experimental group were age between 18 and 65 years, participants of both genders, medical diagnosis of an MCA stroke through magnetic resonance imaging and neurologist’s report, absence of other previous neurological pathologies, being in a subacute (from 15 days to 1 year post-injury) or chronic period of the disease (after 1 year) according to the criteria of the Instituto del Mayor y Servicios Sociales (IMSERSO) model of care for people with brain damage, undergoing rehabilitation treatment at the time of assessment and a score ≥ 24 points on the Mini-Mental State Examination (MMSE). Subjects in the control group met the same criteria, except for the presence of some neurological pathology. 

All participants were informed of the study prior to inclusion and gave informed consent to participate. This study was approved by the Research Ethics Committee of the Universidad Rey Juan Carlos, following the ethical principles for medical research in human beings of the Declaration of Helsinki.

### 2.2. Measures

All the subjects’ data were collected by a single investigator by conducting a clinical history that included information about age, gender and education level; anamnesis; functionality; and EFs. Each participant was evaluated by the following parameters: 

**Functional Assessment Measure (FIM+FAM)** [22], a multidimensional scale that assesses the degree of functional dependence for ADLs. It consists of 30 items, 18 from the FIM (Functional Independence Measure) and 12 from the FAM (Functional Assessment Measure). The total score is obtained by adding the scores of the 30 items. Each of the items has a minimum score of 1 (the functional dependence index) and a maximum score of 7 (the functional independence index). The maximum functional independence level corresponds to a score of 21,016.

**Lawton and Brody’s instrumental activities of daily living scale** [23], which assesses a person’s ability to perform tasks involving the handling of common utensils and everyday social activities. It consists of eight items, and each item has several possible answers to which a numerical value 0 or 1 is assigned. The final score is the sum of the value of all the answers and ranges from 0 (maximum dependence) to 8 (total independence). Higher scores indicate greater independence of the subject.

**The Trail Making Test** (TMT), one of the most widely used neuropsychological instruments. It was designed to assess processing speed and cognitive flexibility. It consists of two parts, Part A and Part B. Both parts of the TMT consist of 25 circles distributed on a sheet of paper. It is scored by the time it takes to perform each of the parts separately. The TMT is scored based on both completion time and error count, where longer completion times and more errors indicate greater impairment [24]. 

**The Zoo Map Test**, one of the subtests of the Behavioural Assessment of Dysexecutive Syndrome (BADS). In the Zoo Map Test, the subject is asked to show how they would visit a series of locations determined on a map of a zoo. There are two versions (Version 1 and 2). The scoring method was designed so that a score profile could be calculated for each test with a range of values from 0 to 4. For each version of the test, the number of errors made is subtracted from the sequence score on the answer sheet. These scores are summed to obtain an overall sequence error score that should not exceed 16 points, in this research we used the direct scores and not the ones taken from the profile [25].

**Modified Rankin Scale** [26], which assesses the level of disability or dependence of people who have had a stroke. It subjectively assigns a score between 1 (no disability) and 5 (high level of disability) regarding the level of independence and uses as a reference the level of independence prior to the stroke. Higher scores reflect a high level of disability.

**The Tower of Hanoi**, which examines the subject’s ability to solve complex problems. We used the computerized version, which consists of a free downloadable computer program that simulates the real size of the Tower of Hanoi. The subject must move the disks according to the instructions described and makes two attempts with three disks and two attempts with four disks. The examiner scores the number of errors and the program indicates when the subject has finished and the total execution time, which is also scored by the examiner; we used both scores for this research [27].

### 2.3. Statistical Analysis 

The estimated effect size for the main outcome measures established in the present work was 0.60. Considering a power of the statistical test of 0.90 and an alpha error of 0.05 for the comparison of means for independent samples, a minimum of 52 subjects were required for the present work according to the G*Power software (V.3.1.9).

Normality analysis of clinical scores was performed using the Shapiro-Wilk test. The U-Mann–Whitney test was used to determine whether significant differences existed between groups. A statistical threshold of *p* ≤ 0.05 was considered significant. 

Nonparametric tests (Spearman correlation) were used to detect significant means and correlations because the samples violated the statistical normality, considering a value of *p* < 0.05 as statistical significance, with a confidence interval of 95%. Correlation coefficients of 0.00–0.30 were interpreted as poor, those of 0.30 to 0.70 as moderate and those of 0.70 or higher as excellent. The analysis of the variables was performed with the statistical program IBM SPSS Statistics for Windows, V.27.0 (Copyright 2013 IBM SPSS Corp., Chicago, IL, USA).

## 3. Results

In total, 60 participants were initially included, 30 of whom were participants with stroke, and the other 30 were healthy controls matched for age, gender and education level. One of the participants dropped out because they were unable to perform the tests, so we removed the matched control subject as well. The final sample consisted of 58 subjects: 29 subjects with stroke, and 29 healthy control subjects.

The age range of the sample (*n* = 58) was 18 to 65 years (44.07 ± 11.34). Thirty were male, and twenty-eight were female. Regarding the stroke participants, all of them had been diagnosed in the MCA, of whom 11 were ischemic and 18 hemorrhagic. The remaining demographic data can be found in Table 1.

### 3.1. Comparative Analysis

The statistical analysis showed statistically significant differences between control and hemorrhagic stroke participants in all variables. For the comparison between control and ischemic participants, the differences were noted in all variables except for the Tower of Hanoi. There were no significant differences between ischemic and hemorrhagic participants (Table 2).

### 3.2. Correlational Analysis

Correlation analysis for control subjects showed moderate associations between age and TMT A (ρ = 0.453, *p* = 0.14) and TMT B; (ρ = 0.444, *p* = 0.16) and a moderate negative correlation between educational level and TMT B (c = −0.372, *p* = 0.47). No significant correlations were found for the rest of the variables (Zoo Map Test and Tower of Hanoi).

Regarding the correlations of stroke participants, we observed an excellent negative association between mRS and FIM+FAM (ρ = −0.885, *p* < 0.001) and Lawton’s proof (ρ = −0.842, *p* < 0.001). Similarly, a moderate negative and statistically significant correlation was identified between the mRS and the Zoo Map Test (ρ = −0.63, *p* < 0.001). There were also positive and statistically significant correlations between the mRS and the TMT A (ρ = 0.69, *p* < 0.001), TMT B, (ρ = 0.526, *p* = 0.003) and Hanoi Tower 3 (ρ = 0.697, *p* < 0.001). No other significant associations were found for the rest of the variables. 

According to the origin of the stroke, the analysis revealed for ischemic stroke patients (Table 3) excellent negative correlations between mRS and FIM+FAM (ρ = −0.744, *p* = 0.009) and an excellent correlation between age and TMT A (ρ = 0.852, *p* = 0.001). Statistically significant correlations were also observed between the years since diagnosis and the FIM+FAM (ρ = 0.683, *p* = 0.02).

For hemorrhagic stroke patients (Table 3), we observed excellent negative correlations between mRS and FIM+FAM (ρ= −0.942, *p* ≤ 0.001) and Lawton’s proof (ρ = −0.929, *p* ≤ 0.001) and a moderate correlation with the Zoo Map Test (ρ= −0.686, *p* = 0.002). There were also statistically significant correlations between mRS and TMT A (ρ = 0.802, *p* < 0.001) and TMT B (ρ = 0.535, *p* = 0.022), as well as with Hanoi 3 (ρ = 0.769, *p* = 0.001).

## 4. Discussion

The results of this study indicate that there were statistically significant differences between the control group and the experimental group in functional skills and EFs. No statistically significant differences were found in the experimental group between subjects who had sustained ischemic and hemorrhagic stroke. In the correlational analysis of the stroke participants, no significant associations were identified between the variables age, gender and education level in relation to functionality and EF.

Regarding functionality, when we compared the scores of both tests between healthy subjects and subjects with stroke, we observed that subjects with stroke obtained lower scores on the FIM+FAM and the Lawton and Brody scale, indicating greater dependence when performing ADLs. We have found no studies with which to compare the data obtained, because ADL assessments are not usually performed with healthy subjects. However, certain aspects of functionality, such as functional mobility, have been evaluated in persons who have suffered a stroke, comparing them with age- and gender-matched control subjects. In a study that assessed walking ability between stroke and healthy subjects, the authors observed that the former had poorer task performance [28].

Similarly, the comparative analysis of EFs showed statistically significant differences between the control group and the experimental group. With respect to the TMT, the stroke participants’ TMT A and TMT B completion times were significantly longer than those of controls. Measures of TMT completion time are sensitive to the presence of various neurological disorders and can indicate whether both processing speed and cognitive flexibility are impaired in this population [29]. These results are in agreement with those obtained by [30], who suggested that slowed processing speed is the contributing factor to TMT performance differences.

In the case of the control group, a correlational analysis revealed a significant relationship between age and TMT A total score, as well as an association between education level and TMT B score. These results are consistent with those obtained in previous studies, which have indicated that the time to complete the TMT A is affected by age [31,32] and that the time to complete the TMT B is affected by education level, which positively influences subjects younger than 60 years [33].

In the correlational analysis of the stroke participants, no significant associations were identified between the variables gender, age and education level in relation to functionality and executive functions. As in our study, previous scientific literature has identified a lack of relationship between gender and functional aspects [34], which supports the results obtained by Önes et al. [35], who did not identify significant effects of gender on functional outcomes in stroke participants. Regarding age, some studies have explained a negative relationship between increasing age and worsening functional outcomes as being due to increased comorbidity and disability [36], while other authors have suggested that functionality does not depend on the age at which the patient suffered the stroke [33]. Similarly, previous studies have suggested that a high education level may influence better motor and functional recovery outcomes [37]. In relation to EFs, in our study, no statistical relationships were found in the experimental group, although previous studies have indicated that age and education level are related to cognitive functioning [38,39]. However, it is important to emphasize that the impairment of cognitive functions is determined by the state of cognitive reserve, which determines the ability of the person who has suffered a stroke to perform a certain task despite the fact that damage has been observed in the specific brain area responsible for performing that function [40]. In this case, when neuronal damage occurs, there are recruitment and activation of other brain networks to carry out these functions [41]. This ability to compensate for the damage caused is determined by experiences and static factors such as intelligence level, work achievements, or educational level [42,43,44]. Our results might be explained because most of the scientific literature has evaluated the relationship between these variables after evaluating the efficacy of rehabilitation programs in stroke participants. However, we cannot deny that it was surprising that education level did not present a correlation with all the EF outcomes in stroke patients, unlike the findings we found in the control group. These results could be due to several factors. Firstly, there could be heterogeneous levels of education in the experimental group. Secondly, a relationship might be present, but a larger sample size could show this connection. Finally, the assessment tools used in the experimental group should be reinforced with other tests to identify possible connections in future studies. 

The levels of disability assessed with the mRS were significantly associated with the level of functionality and independence in ADLs and EFs. In this sense, the use of functional and independence assessments of ADLs have been commonly used to measure disability after stroke. In our study, the level of disability correlated negatively with the results of the FIM+FAM and the Lawton and Brody test. These results are similar to those obtained by Miki et al. [45], which indicated that higher levels of disability are associated with a higher level of dependency and functional difficulties.

Similarly, in our study, the level of disability was significantly associated with the EFs assessed, which is consistent with previous studies that have identified that deficits in EFs are associated with worse outcomes in functional capacity and quality of life [46,47,48]. In the literature, the percentage of participants with acute stroke who present difficulties in EFs varies from 19% to 75% [49,50,51]. In this regard, in our study, planning skills, which we assessed with the Zoo Map Test, were negatively associated with the level of disability, similar to tests that assess cognitive flexibility and problem-solving skills, which were associated with worse performance and a higher level of disability. In line with these results, Park et al. [52] suggested that stroke severity is one of the factors that shows greater predictive value in relation to the patient’s functional level than other demographic variables that may predict the patient’s functional outcome but are no longer significant when cognitive aspects are included. Specifically, a correlational analysis according to stroke type showed that in the sample of participants with a hemorrhagic stroke, stroke severity contributed to a greater statistical association with the level of functionality and EFs compared to the group of participants with ischemic stroke, which coincides with current evidence suggesting that hemorrhagic strokes have a worse functional and clinical status compared with ischemic ones [53,54].

The information presented in this article has important clinical implications for improving the treatment approach to EFs and ADLs in stroke participants. Knowledge of EFs and their involvement in ADLs is extremely useful in establishing the need for a stronger emphasis on the rehabilitative treatment of stroke participants. Occupations are purposeful and meaningful activities for people, so treatment should focus on the individual’s ability to be able to participate successfully in all areas of occupation. We believe that knowledge of all cognitive abilities that directly affect the performance of ADLs should be considered by all members of the rehabilitation team. In view of the results on the influence exerted by EFs on the performance of ADLs in people with stroke, we propose the need to develop standardized protocols for the assessment of people with stroke so that there is a consensus on the use of scales for the evaluation of EF and ADLs. In this way, a more exhaustive diagnosis can be made in relation to the limitations of the patient to improve treatment. This study adds to the scarce literature available on the influence and relationship of sociodemographic factors on the executive functions and level of functional independence of stroke patients. In addition, to our best knowledge, it is the first to analyze the influence of education level in patients with hemorrhagic stroke. Future studies should be conducted to analyze whether improvements in cognitive outcomes could be related to better ADL performances and to corroborate, with larger sample sizes, our findings in terms of age, gender and level of education in the stroke population compared to healthy subjects. Finally, identifying sociodemographic factors in the stroke population linked to EF and functionality might improve practice guidelines in post-stroke patients. 

This study presents several limitations. First, our sample size was small. In the correlational analysis of the stroke participants, no significant associations were identified between the variables gender, age and education level in relation to functionality and EF, so the sample should be expanded. On the other hand, no specific EF assessment tool was available for people with stroke, so we used the most clinically relevant ones. Finally, we cannot extrapolate our findings to all stroke patients, so our results must be read with caution.

## 5. Conclusions

People who have suffered a stroke of the right MCA present impairments in EFs, resulting in a worse performance of ADLs compared to healthy subjects matched for age, gender and education level. No statistically significant differences were found in the experimental group between subjects who had sustained ischemic and hemorrhagic strokes in terms of functionality and EF. In the correlational analysis of the stroke participants, no significant associations were identified between the variables age, gender and education level in relation to functionality and EF. 

## Figures and Tables

**Table 1:** Sociodemographic information for stroke participants and healthy controls.

Variable		Stroke Participants (*n* = 29)		Controls (*n* = 29)
		Hemorrhagic (*n* = 18)	Ischemic (*n* = 11)	
Gender		12 women, 6 men	2 women, 9 men	14 women, 15 men
Age		42.44 ± 13.47	46.73 ± 6.28	44.07 ± 11.34
Years Since Stroke (months)		11.56 ± 9.02	21.82 ± 17.42	-
Education Level	No studies	1	0	1
	General education	2	4	6
	High school	5	4	9
	Advanced vocational training	2	1	4
	University degree	8	2	9

**Table 2:** Comparative analysis of measurement scales.

Scale	Control IR, M (SD)	Stroke IR, M (SD)	*p*	Ischemic ^a^ IR, M (SD)	*p*	Hemorrhagic ^b^ IR, M (SD)	*p*	Hemorrhagic vs. Ischemic *p*
mRS	---	3.27 (4)	---	3.25 (3)	---	3.28 (4)	---	0.945
FIM+FAM	210 (0)	150.72 (54)	<0.001	161.91 (35)	<0.001	143.89 (69)	<0.001	0.361
Lawton	8 (0)	2.93 (3)	<0.001	2.55 (1)	<0.001	3.17 (4)	<0.001	0.889
Zoo Map Test	13 (3)	6.1 (10)	<0.001	5 (11)	<0.001	6.78 (9)	<0.001	0.998
TMT A time	32.59 (17)	95.07 (68)	<0.001	73.45 (53)	<0.001	108.28 (90)	<0.001	0.668
TMT B time	72.97 (47)	143.38 (94)	<0.001	158.18 (81)	<0.001	134.33 (104)	<0.001	0.494
Hanoi 3 time ^b^	40.64 (40)	71.11 (54.88)	0.006	51.07 (43)	0.165	80.47 (55.5)	0.017	0.461
Hanoi 4 time ^b^	78.91 (59.75)	138.75 (97.38)	0.001	128.36 (104)	0.072	143.6 (98.5)	0.008	0.932

Note: mRS = Modified Rankin Scale; FIM+FAM = Functional Assessment Measure; Lawton = Lawton and Brody’s (1969) IADL scale; TMT = Trail Making Test; Hanoi = Tower of Hanoi. ^a^ *n* = 26 (11 ischemic, 15 hemorrhagic). ^b^ *n* = 22 (7 ischemic, 15 hemorrhagic).

**Table 3:** Correlation analysis for stroke participants.

Ischemic Correlation	Gender			Age			Level of Education			Years Since Diagnosis			mRS		
	ρ	CI	*p*	ρ	CI	*p*	ρ	CI	*p*	ρ	CI	*p*	ρ	CI	*p*
FIM+FAM	0	−0.559 to 0.599	1	−0.082	−0.649 to 0.545	0.811	−0.11	−0.666 to −0.524	0.748	0.683	0.141 to 0.91	0.02	−0.744	−0.929 to −0.26	0.009
Lawton	0.039	−0.574 to 0.624	0.91	−0.472	−0.835 to 0.178	0.143	0.386	−0.278 to 0.8	0.241	0.333	−0.333 to 0.778	0.333	−0.479	−0.838 to 0.169	0.136
Zoo Map Test	0.149	−0.495 to 0.687	0.662	−0.364	−0.791 to 0.302	0.27	−0.196	−0.703 to 0.458	0.564	−0.178	−0.703 to 0.472	0.601	−0.588	−0.878 to 0.018	0.057
TMT A time	−0.298	−0.762 to 0.368	0.373	0.852	0.516 to 0.961	0.001	−0.272	−0.749 to 0.392	0.418	0.374	−0.291 to 0.795	0.258	0.372	−0.293 to 0.795	0.26
TMT B time	−0.596	−0.881 to 0.006	0.053	0.574	−0.039 to 0.873	0.065	−0.33	−0.776 to 0.336	0.322	0.164	−0.483 to 0.695	0.63	0.362	−0.303 to 0.79	0.274
Hanoi 3 time	0	−0.753 to 0.753	1	−0.036	−0.768 to 0.737	0.939	−0.538	−0.919 to 0.362	0.213	−0.198	−0.828 to 0.652	0.67	0.558	−0.336 to 0.923	0.193
Hanoi 4 time	−0.408	−0.888 to 0.498	0.363	0.429	−0.479 to 0.893	0.337	−0.299	−0.859 to 0.586	0.515	0.234	−0.63 to 0.839	0.613	0.558	−0.336 to 0.923	0.193
**Hemorrhagic correlation**	**Gender**			**Age**			**Level of Education**			**Years Since Diagnosis**			**mRS**		
	**ρ**	**CI**	***p***	**ρ**	**CI**	***p***	**ρ**	**CI**	***p***	**ρ**	**CI**	***p***	**ρ**	**CI**	***p***
FIM+FAM	−0.25	−0.642 to 0.246	0.317	−0.187	−0.601 to 0.307	0.457	−0.222	−0.624 to 0.273	0.376	0.054	−0.414 to −0.508	0.831	−0.942	−0.979 to −0.848	<0.001
Lawton	−0.244	−0.638 to 0.252	0.329	−0.163	−0.585 to 0.329	0.518	−0.168	−0.589 to 0.324	0.505	0.014	−0.456 to 0.478	0.955	−0.929	−0.974 to −0.816	<0.001
Zoo Map Test	−0.171	−0.591 to −322	0.497	−0.258	−0.647 to 0.237	0.302	0.295	−0.199 to 0.669	0.234	−0.102	−0.543 to 0.383	0.686	−0.686	−0.873 to −0.322	0.002
TMT A time	0.363	−0.125 to 0.709	0.363	0.254	−0.242 to 0.644	0.309	0.098	−0.387 to 0.54	0.698	−0.015	−0.479 to 0.456	0.954	0.804	0.539 to 0.924	<0.001
TMT B time	0.182	−0.311 to 0.598	0.182	0.147	−0.343 to 0.574	0.561	0.272	−0.223 to 0.656	0.274	0.027	−0.445 to 0.488	0.915	0.535	0.091 to 0.802	0.022
Hanoi 3 time	0.229	0.321 to 0.663	0.411	0.05	−0.474 to 0.548	0.859	0.265	−0.286 to 0.684	0.265	0.165	−0.379 to 0.624	0.556	0.769	0.424 to 0.919	0.001
Hanoi 4 time	−0.295	−0.701 to 0.256	0.286	0.182	−0.364 to 0.635	0.515	−0.074	−0.565 to 0.456	0.794	−0.246	−0.673 to 0.305	0.377	0.187	−0.359 to 0.638	0.504

Note: CI = 95% confidence interval; mRS = Modified Rankin Scale; FIM+FAM = Functional Assessment Measure; Lawton = Lawton and Brody’s IADL scale; TMT = Trail Making Test; Hanoi = Tower of Hanoi.

## Data Availability

Data are not available due to privacy and ethical restrictions.
